# Gastric Mass: Intramural Hematoma

**DOI:** 10.7759/cureus.18926

**Published:** 2021-10-20

**Authors:** Kathryn Schwalbe, Abhiram Kondajji, Matthew T Allemang

**Affiliations:** 1 Surgery, Cleveland Clinic- South Pointe Hospital, Warrensville Heights, USA; 2 Surgery, Cleveland Clinic - South Pointe Hospital, Warrensville Heights, USA

**Keywords:** gastric bleed, gastric tumor, mass, gastric ulcer, partial gastrectomy

## Abstract

Gastric masses can be challenging to diagnose pre-operatively due to their heterogeneity in presentation and work-up. One must be cautious that a seemingly benign mass may be malignant and vice versa. Some of the more common gastric masses include peptic ulcer, adenocarcinoma, and gastrointestinal stromal tumour. These diagnoses have vastly different management strategies despite similar presentations. The case presented here is an example of this management, highlighting a patient with a gastric bleeding mass initially thought to be a gastrointestinal stromal tumour. However, on final pathology, the mass was determined to be benign, an ulcerated hematoma.

## Introduction

Gastric masses can be difficult to accurately diagnose due to a widespread differential with similar radiologic and endoscopic characteristics ranging from benign to malignant conditions. The most common symptom experienced by patients with gastric masses is gastrointestinal bleeding; however, masses are more commonly asymptomatic. If a mass is identified on initial evaluation for the bleed with an esophagogastroduodenoscopy (EGD), further investigation with computed tomography (CT) scan and surgical intervention may be warranted.

Some examples of common diagnoses that cause gastric masses include peptic ulcer disease (PUD), gastric adenocarcinoma, and gastrointestinal stromal tumour (GIST). Peptic ulcer disease is the cause of the majority of upper gastrointestinal bleeding in the United States. The bacteria Helicobacter pylori, non-steroidal anti-inflammatory medications (NSAIDs), and Aspirin are identified as the cause in 80% of cases [1]. H. pylori are also implicated in developing 80% of gastric adenocarcinoma, which is the most common gastric malignancy [2]. GIST is a rare submucosal tumour that may be located anywhere within the gastrointestinal tract. They are usually located in the stomach and undetected until 5cm in size [3]. Treatments for the abovementioned diagnoses include medical management, surgery, chemotherapy, or radiation [1,4,5]. When the mass is considered potentially malignant, as is the case with adenocarcinoma and GIST, the surgical treatment goal is negative margins without disruption of the capsule. Surgery can be successful via either a laparoscopic or open approach [4,5].

The case to follow is an example of such a challenge. In brief, our patient presented with an upper gastrointestinal bleed, underwent endoscopy and imaging, which suggested GIST as the diagnosis and underwent appropriate surgical treatment only to discover that the mass was a large intramural hematoma on the final pathology report.

## Case presentation

Patient Presentation

**Figure 1:**
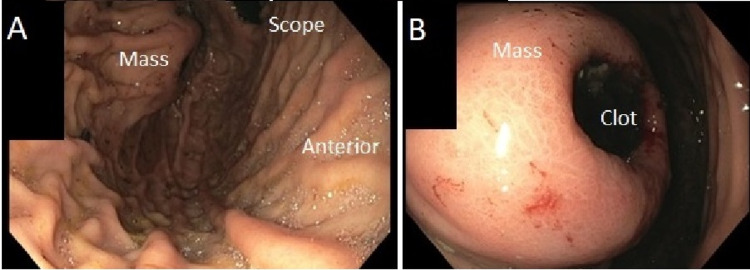
Endoscopic appearance of the approximately 5cm ulcerated submucosal mass located along the proximal posterior fundus. A) Overall view of the mass. B) Close-up view of mass.

The patient is a 43-year-old male with end-stage renal disease who presented to our community hospital with hematemesis. He underwent an EGD with our gastroenterology colleagues, which found an approximately 5cm proximal posterior gastric wall mass with ulceration and central clot (Figure 1). There was no active bleeding. The clot was easily unroofed and biopsied, and hemostasis was subsequently obtained. At this point, the general surgery team was consulted. Of note, the patient had stopped taking Warfarin for catheter-related deep vein thrombus three days before his episode of hematemesis and his INR level was subtherapeutic on presentation.

Evaluation

**Figure 2:**
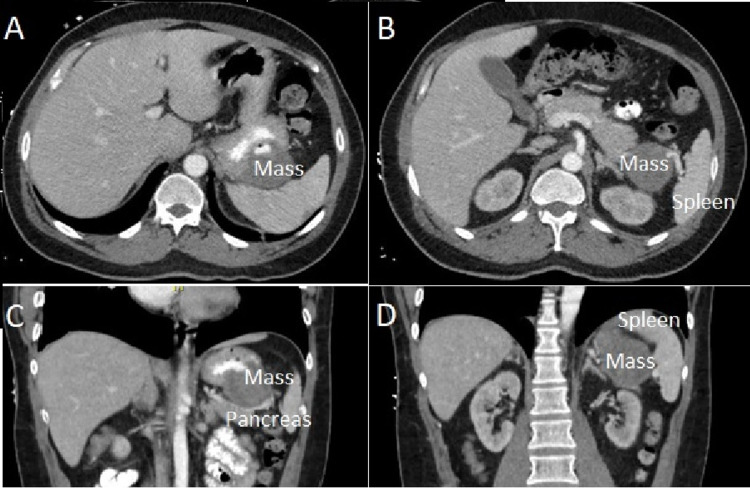
CT imaging of the gastric mass. A) Axial cut of the superior abdomen, demonstrating ulceration of the mass and proximity along the superior posterior gastric wall. B) Axial cut of the superior abdomen, demonstrating proximity of the mass to the splenic hilum. C) Coronal cut of the abdomen, demonstrating proximity of mass to pancreatic tail. D) Coronal section of the abdomen demonstrating proximity to the splenic hilum.

Due to the large size of the identified mass, the patient was at increased risk for severe recurrent upper gastrointestinal bleeding and malignancy. This prompted a relatively short timeline from diagnosis to surgical intervention. A computed tomography (CT) scan of the chest without intravenous (IV) contrast; and a CT scan of the abdomen and pelvis with oral and IV contrast was obtained to assess for potential metastases (Figure 2). The abdominal imaging demonstrated a 5.0 cm heterogeneous mass involving the posterior wall of the gastric fundus without obvious extension into nearby tissues such as the spleen, splenic vessels, or pancreas. There were no findings suggestive of metastasis in the chest nor pelvis. The EGD tissue biopsy of the ulcer demonstrated mucosal gastritis. There were no signs of dysplasia nor H. pylori.

Despite the high likelihood that a gastric ulcer is benign, there is a 5-11% chance that the ulcer is malignant. Both benign and malignant ulcerations have associations with H. pylori. Having a benign biopsy without H. pylori present decreased the chance of this mass being a peptic ulcer or adenocarcinoma [4,6]. In gastric adenocarcinoma, the macroscopic appearance and radiology imaging did not suggest this malignancy [4]. Endoscopy for a GIST tumour often shows a solitary ulcerated submucosal mass. CT imaging of a GIST most commonly shows a well-circumscribed mass that may be heterogeneous due to haemorrhage or necrosis [3]. A submucosal tissue biopsy such as FNA or core needle is indicated in cases where the diagnosis is not specific, the mass is considered benign, or the patient may benefit from pre-operative chemotherapy to make the mass operable [3,7]. However, an FNA usually does not have enough tissue for a reliable diagnosis, and a CT-guided core needle biopsy would have been high risk due to the location of this mass [3]. 

The gastrointestinal stromal tumour was considered the most likely diagnosis based on the pre-operative endoscopic and CT imaging findings of a single ulcerated submucosal mass with heterogeneous contrast enhancement. A pre-operative submucosal tissue sample via either FNA or core needle was considered low-yield and high risk. Our team, therefore, recommended surgical resection of the mass to diagnose and treat this patient definitively. 

Surgery

The pre-operative plan for this patient was to attempt a laparoscopic wedge resection, as the mass was expected to be non-invasive and along the more significant curve of the stomach with plenty of gastric wall remaining. However, we were prepared to change course if our pre-operative assessment was discordant with our direct intra-operative evaluation.

**Figure 3:**
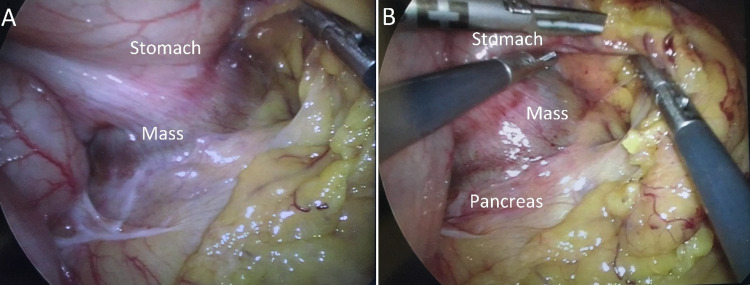
Intra-operative appearance of the gastric mass. A) Adhesions between the posterior gastric mass and spleen. B) Adhesions between mass and pancreatic tail.

During initial laparoscopic visualization, the gastric mass was adherent to both the splenic hilum and the distal tail of the pancreas (Figure 3). This raised suspicion for malignancy, and the surgical goal was not just to remove the mass but also to achieve a negative margin resection. No peritoneal implants were identified. During dissection of the mass from the splenic hilum, bleeding was encountered originating from the hilum, and the decision was made to convert to an open procedure. The mass was noted to be so densely adherent to the distal tail of the pancreas that a safe plane could not be found, prompting a distal pancreatectomy. Our gastroenterology colleagues performed an intra-operative EGD, which demonstrated sufficient lumen remaining in the stomach to allow the mass wedge resection. The patient ultimately underwent an en bloc wedge resection of the pancreas’s gastric mass, spleen, and distal tail. Post-operatively, he developed a small asymptomatic pancreatic leak which was managed conservatively and otherwise recovered well.

Pathology

**Figure 4:**
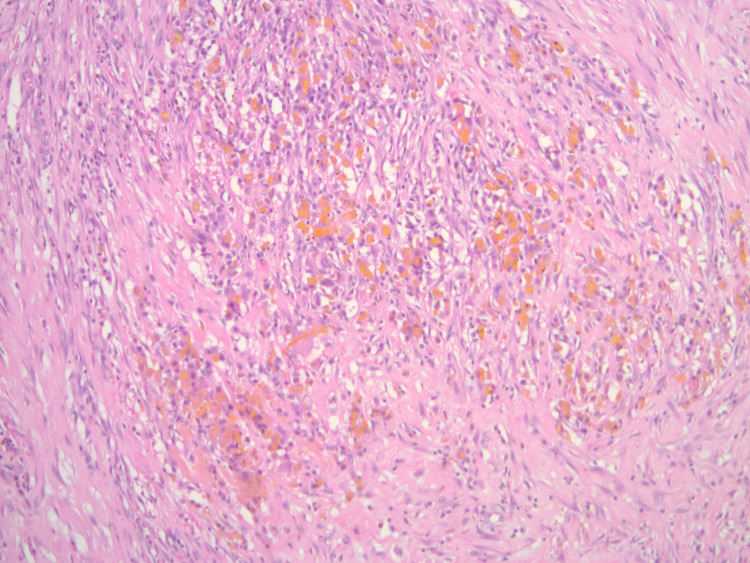
Pathology slide demonstrating recent haemorrhage at 100x magnification with hematoxylin and eosin stain, suggestive of hematoma—courtesy of Dr Sebouh Setrakian MD.

Following review by multiple pathologists, the final pathology report described the mass as hemorrhagic ulceration (Figure 4). No malignancy or other histologic cause for the ulceration was identified.

## Discussion

The case presented here provides an example of the challenges associated with diagnosing a gastric mass in the pre-operative setting. More common diagnoses such as peptic ulcer disease, adenocarcinoma, and GIST have overlapping endoscopic and radiologic findings with rare diseases. Sometimes an ulcerated submucosal mass is not a GIST even when macroscopic appearance seems convincing. Other submucosal tumours can include those originating from any tissue present: lymphatic, muscular, nervous, mesenchymal, or vascular structures [7].

Symptomatic intra-mural gastric hematomas are exceedingly rare, with fewer than 40 other cases available in the literature and fewer than 15 treated with surgery [8,9]. Because of their rarity, treatment recommendations are not standardized [8]. The most common causes of intramural gastric hematoma include coagulopathy (53%), aneurysm (15%), PUD (11%), spontaneous (11%), and miscellaneous other (7%). Of the intramural gastric hematomas attributed to coagulopathy, 15% are caused by therapeutic anticoagulation. These cases may be managed conservatively with reversal of the anticoagulant [9,10].

## Conclusions

Gastric masses can be challenging to treat because of their diagnostic challenges and may have unexpected intra-operative findings. Deciding which treatment is best for your patient at the time of presentation depends on a thorough evaluation while recognizing that even our current best technology is imperfect. Surgical intervention can still be warranted before a final pathologic diagnosis to palliate a patient’s symptoms. The treating surgeon should be prepared to alter the operative treatment relative to intra-operative findings.
